# Experiences of Stigma and Discrimination among Caregivers of Persons with Schizophrenia in China: A Field Survey

**DOI:** 10.1371/journal.pone.0108527

**Published:** 2014-09-26

**Authors:** Yi Yin, Weijun Zhang, Zhenyu Hu, Fujun Jia, Yafang Li, Huiwen Xu, Shuliang Zhao, Jing Guo, Donghua Tian, Zhiyong Qu

**Affiliations:** 1 School of Social Development and Public Policy, Beijing Normal University, Beijing, P. R. China; 2 Office of Director, Ningbo Kangning Hospital, Ningbo, Zhejiang, P. R. China; 3 Guangdong Mental Health Center, Guangdong General Hospital, Guangzhou, Guangdong, P. R. China; 4 Department of Medical Affairs, Peking Union Medical College Hospital, Beijing, P. R. China; 5 Department of Public Health Sciences, University of Rochester School of Medicine & Dentistry, Rochester, New York, United States of America; 6 School of Public Management, Yunnan University of Finance and Economics, Kunming, Yunnan, P. R. China; 7 Department of Sociology, Huazhong University of Science and Technology, Wuhan, Hubei, P. R. China; University of Geneva, Switzerland

## Abstract

In China, caregivers for family members with schizophrenia play an important role in treatment and recovery but may experience stigma and discrimination simply because of their family relationship. The object of this study was to measure the degrees and correlates of stigma and discrimination experiences among this group. Four hundred twenty-seven caregivers participated in this hospital-based and cross-sectional study in *Ningbo* and *Guangzhou*, China. Data were collected by trained interviewers using fixed questionnaires. Stigma and discrimination experiences were measured by the Modified Consumer Experiences of Stigma Questionnaire (MCESQ). Caregivers’ social support was measured by the Social Support Rating Scale. Parametric analysis, nonparametric analysis and multivariate linear regression were used. The mean (*SD*) score of MCESQ was 2.44(0.45), 2.91(0.71) for stigma experiences and 1.97(0.37) for discrimination experiences on a five-point score (“1 = never” and “5 = very often”). Approximately 65% of caregivers reported that they tried to conceal their family members’ illness, and 71% lacked the support of friends. The experience of stigma was significantly negatively associated with the perceived social support of caregivers (standard β = −0.2,*p*<0.001). Caregivers who were children of the patients experienced fewer stigmas than other (standard β = −0.18, * p*<0.001). Urban residence (standard β = −0.12, *p*<0.01) and patients did not complete primary school education (standard β = −0.13, *p*<0.01) were negatively related with stigmas. In addition, stigma and discrimination was more experienced in *Zhejiang* than in *Guangdong* (*p<*0.05). In conclusion, this study performed that caregivers of people with schizophrenia in China experienced general stigmas and rare discrimination and found the relations with social support, kinship, patient’s educational level and regional differences. More interventions and supports should been given to caregivers who are lack of social support, who live in rural area and who are the patients’ parents, spouses or siblings.

## Introduction

Schizophrenia is one of the most severe mental illnesses in the world. This illness creates burdens for families and affects the quality of life of relatives [1]–[2]. Patients also suffer from stigmas, discrimination (the behavioral aspect of stigma), as well as exclusion from community life in housing, education, employment and social and family relationships [3]–[5]. At the same time, stigmas and discrimination are also common among families, who share similar beliefs about inferiority and may feel a lessened sense of humanity [6]. Other research also has shown that family members of serious mental health consumers, especially caregivers of patients with schizophrenia, often encounter stigma and discrimination [4], [7]–[10]. Family stigma negatively impacts caregivers and persons with schizophrenia, creating burdens, enhancing stress and affecting the quality of life [11].

One explanation for stigmas and feelings of guilt is that the origin of illness can be traced to family [9]. In addition, social and cultural factors can enhance “genetic risk”. Karamlou and Mottaghipour’s interviews in Iran showed that cultural differences might influence the experience of stigma in families of psychiatric patients in areas such as concealment, limitation in work and education, genetic attributions, traditional beliefs in society about patients, gender differences and so on [12]. Snowden and Yamada also stressed that one source of stigma could not replace another; it was therefore important to pay more attention to gender, race and immigrant identities in the stigmatized family [13]. In fact, in traditional Chinese society, there is a cultural expectation that families should stay at the hospital or at home to care for patients, leading to much more involvement in the care of people with severe mental illness [14]–[16]. Therefore, illness is a family issue rather than an individual problem [17].

Existing research has discussed the degree to which stigma is experienced or perceived among caregivers in China. For example, Phillips et al. found that 26% of 952 family members of persons with schizophrenia in five sites around China faced a moderate to severe degree of stigma, based on interviews [18]. Lee and colleagues performed a study using a focus group in Hong Kong suggesting that approximately 40% of patients with schizophrenia reported that their family members were unfairly treated because of the patients’ illness, and over 60% of their family members and partners chose to conceal their relationship [19]. Research by Gao and his colleagues indicated that 56% of relatives of patients with schizophrenia kept the disease secret in Beijing, China [20].

Some research worldwide has discussed factors which are related with stigma and discrimination experienced or perceived by family caregivers of patients with severe mental illness. First, different social-demographic factors affect perceptions of stigma. Urban residents suffered a higher level of stigma compared with those in rural areas [18], [21]. Furthermore, highly educated families are more sensitive to stigma [18], [22]. For example, Shibre and colleagues found that the urban residents whose age were over 45, perceived more stigma in rural Ethiopia [23]. In addition, Charles, Manoranjitham and Jacob found a significant association between stigma scores of patients and relatives in south India and that the relatives’ stigma scores were significantly associated with male gender, literacy, rural residence, and belief that illness was due to karma and total patient stigma scores [24]. Second, the clinical history of patients can also affect the degree of stigma. A long duration of illness will increase stigma [25], and the diagnosis of an illness within 6 months may lead to increased avoidance by others [22]. Finally, the psychological states and characteristics of caregivers are other related factors. Magaña et al. discovered that stigma was significantly correlated to depression in Latino family caregivers of persons with schizophrenia [26]. In Ethiopia, it was found that caregiver’s self-stigma levels were significantly related to their knowledge and the belief in supernatural explanations for the disease [21]. Additionally, high levels of expressed emotion can cause greater stigma [18].

There has been little research into stigma among Chinese family caregivers, although Chinese family caregivers of mentally illness relatives are usually more involved in clinical treatment and care due to traditional family values [13]. Existing research has proposed that stigma is associated with such threats as “genetic contamination” and “losing face” in the Chinese cultural context, but little research has explicitly identified and tested the effects of this threat to family lineage among Chinese groups [27], [28]. Nearly all existing research about stigma and schizophrenia has used quantitative methods with a lack of systematic assessments; furthermore, little is known about the specific stigma and discrimination experiences of the caregivers of patients with schizophrenia. Another limitation of prior studies is that their sample selection was based on typical individuals rather than on large-sample, without exploring the breadth and depth of the problem [11].

This article will investigate the stigma and discrimination experienced by caregivers in their daily life, such as in education, housing, job hunting and other social activities in China. In particular, we will explore the correlation between the experience and possible associates, like social-demographic variables and caregivers’ perceived social support.

## Methods

### 1. Study design

This study is a cross-sectional and hospital-based survey conducted in *Guangzhou*, and *Ningbo*. *Guangzhou* is also known as *Canton* and is the capital of *Guangdong* Province; *Ningbo* is the second largest city in *Zhejiang* Province. In 2010, there were 12.7 million resident population in *Guangzhou* and 7.6 million in *Ningbo* according to the sixth Nationwide Population Census. The survey conducted in three mental health hospitals in *Guangzhou* affiliated with Guangdong Mental Health Center and one in *Ningbo* under Ningbo Mental Health Center from June 2012 to July 2012. These centers undertook the main responsibilities for service providing and technique guidance, in which approximately 70% local schizophrenia patients acquired direct and indirect treatment and recovery service.

### 2. Participants and sample procedures

Before the survey, a focus group discussion was conducted to determine the definition of caregivers and sampling procedures in which psychiatrists, researchers, family members participated. According to the purpose and design of the study, only one primary caregiver for each patient was surveyed and was defined as: (1) who was regarded by patient as main caregiver; (2) whose self-report care workload percentage among all family members who gave care to the patient should be more than 30%; (3) whose care duration was more than one-year except for patients diagnosed in 2012. And including standard was: (1) family members of people with schizophrenia; (2) age above 18 years old; (3) outpatients or inpatients taken care of by participants had to meet the Chinese Classification of Mental Disorders 3rd edition (CCMD-III) criteria for schizophrenia. CCMD- III is a clinical guide in China that is widely used to diagnose mental disorders. It is based on International Statistical Classification of Diseases and Related Health Problems 10th Revision (ICD-10) and a few variations exist because of cultural differences.

The participants came from two parts: First, the primary caregivers of all the inpatients during the period of investigation were invited by our team and the nurses were responsible to get in touch with them; second, caregivers who accompanied with the outpatient to hospital in the morning during the two weeks also were invited to participate in this research.

Sample size calculation was based on a conservative estimate 50% (prevalence of stigma), while a 5% error rate was adopted in the calculation. The study would need to have a sample of 384 participants at least. Finally, there were a total of 427 samples for analysis in this study.

Cooperative personnel were recruited after being fully informed about the research by trained investigators (including graduate students, nurses and intern doctors) in our research team. The investigators conducted face-to-face structured interviews using pretested questionnaire with caregivers in Mandarin or local dialects.

### 3. Measurements

**Stigma and discrimination** were measured by the Modified Consumer Experiences of Stigma Questionnaire (MCESQ), which assessed negative reactions experienced because of relatives with schizophrenia in the past month [29]–[30]. The questionnaire was modified, and one item about unequal treatment about health insurance was not measured for this study, given that the health insurance in China was arranged in state-level which combined with New Rural Cooperative Medical Scheme, Urban Residents’ Basic Medical Insurance and Urban Employee Basic Medical Insurance, and the medical reimbursement depended on policy at that time. The scale used in our study had 18 items and 2 subscales (stigma experience and discrimination experience) that assessed the degree to which an individual had perceived negative social actions. The “stigma” scale measured the degree to which caregivers dealt with negative attitudes from others because of their relatives’ severe mental illness. For example, “I have worried that others will view me unfavorably because my family member receives psychiatric treatment.” The “discrimination” scale measured whether caregivers experienced discrimination in working, housing, participation social activities, etc. because of the fact that they were caring for relatives with schizophrenia. One of the examples was “I have been avoided indicating on written applications (for jobs, licenses, housing, school, etc.) that my family received psychiatric treatment for fear that information would be used against me or my family.” Each item was rated on a five-point Likert scale that was anchored at “1 = never” and “5 = very often”. A total score of caregivers’ stigma and discrimination experience was computed by summing up the individual items after reversely coding reverse scoring item. A higher score therefore indicated higher stigma and discrimination experience. The efficacy of this scale in measuring experiences of stigma has been previously demonstrated [31]. The review of the literature indicated that the average coefficient for internal consistency for the CESQ was 0.78 [32]. The coefficient for internal consistency in this sample was Cronbach’s α = 0.67.

**Social support** was measured using the Social Support Rating Scale (SSRS), developed and modified by Xiao [33]. This scale consists of 10 items and three major categories that measured subjective support, objective support and the use of differing degrees of support. For example, “How many close friends you have that can give you support and help?” was asked to measure the support from friends. The highest possible score is 66, with a higher score signifying more social support. This scale is widely used in China with good reliability and validity. The Cronbach’s α in this study was 0.724.

**Demographic and background information** collected in this study for caregivers and their relatives included age, gender, marital status (single, married, divorced/widowed), kinship (spouse, child, parent, sibling, other), education level (≤primary school, middle school, high school, college and above), province of residence (*Guangdong*, *Zhejiang*), settings (rural, urban), employment status (employed, unemployed, retired), family income (*yuan* per year), duration of caring (year), and other items related to the illness, such as the duration of disease and phase of illness (prodromal phase, acute phase, remission).

### 4. Data analysis

Data was double-entered into EpiData version 3.1 (EpiData Association, Odense M, Denmark). Then, data was imported into STATA version 12.0 for Windows (Stata Corp, College Station, Texas USA) for data completeness, outlier clearance and data analyses. Descriptive analyses were used to report stigma and discrimination status, degree of social support, social-demographic status and the status of the patient’s illness. Normality of the stigma experience score, discrimination experience score and total MCESQ score were checked using Skewness-Kurtosis test. Scores of stigma experience and total MCESQ scores met the standard of normal distribution. Since the discrimination score was not normally distributed and it could not be normal after logarithmic transformation and exponential transformation, non-parametric test and robust regression were used for the analysis of discrimination score. Therefore, (1) Pearson analysis, T-test and ANOVA was used to identify the relationships among stigma experience, social support and other factors; (2) in terms of discrimination experience, bivariate analyses were conducted using Spearman analysis, Ranksum-test and Kruskal-Wallis test; (3) bivariate test about MCESQ score was the same as that of stigma score; (4) multiple linear regressions were used to analyze the associates of the stigma experience score and total MCESQ score. (5) robust regression was conducted to analyze related factors of discrimination experience score. Finally, the presence of multi-collinearity was also checked.

### 5. Ethics statement

The study was approved by the Institutional Review Board of the School of Social Development and Public Policy at Beijing Normal University. Approved verbal informed consent was used rather than signature or fingerprint one because participants regarded the latter as sensitive issues in local culture and it might lead to high refusal rate. The consent was read by the investigators before starting the interviews to keep every study participant be informed of the research purpose, procedures, potential risks and benefits of participation, confidentiality protection, the right to refuse or withdraw and so on. The interview was started or stopped at the participant request. Our ethics committee approved this consent procedure.

## Results

### 1. Socio-demographic characteristics

Table 1 and Table 2 summarize the social and demographic characteristics of caregivers. 229(53.6%) caregivers in *Guangdong* and 198(46.4%) caregivers in *Zhejiang* were included in this study. The average age of caregivers was 50.88 years (min = 20, max = 83, standard deviation [*SD*] = 12.62), about half (52.0%) were male. In terms of family kinships, 54.6% of primary caregivers were parents of patients, 26.5% were spouses, 10.5% were children, 6.3% were siblings and 2.1% were other relatives. Nearly three-quarters (74.3%) had less than nine years of education. Only 17.6% were unemployed. 53.4% of caregivers lived in rural areas. Average of caring year was 7.26(*SD* = 3.37). Family income was 46,551 RMB (about 7,389 U.S. dollars, 1 USD ≈ 6.3 RMB, June 2012) per year on average. The score of social support was range from 14 to 51 and mean score was 28.76 (*SD* = 7.08).

**Table 1:** Correlations between continuous variables of caregivers and patients with the mean of stigma scores, mean of discrimination scores and mean of MCESQ scores.

Variable			Mean of stigma		Mean of discrimination		Mean of MCESQ	
	Mean	*SD*	r	*p*	r	*p*	r	*p*
**Caregiver**								
Age(year)	50.88	12.62	0.12	(0.012)	− 0.03	(0.516)	0.08	(0.109)
Care duration(year)	7.26	3.37	0.03	(0.572)	−0.05	(0.315)	0.02	(0.699)
Family income( 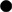 )	46551	50265	−0.07	(0.128)	−0.01	(0.785)	−0.07	(0.130)
SSRS scores	28.76	7.08	−0.24	(<0.001)	−0.07	(0.167)	−0.21	(<0.001)
**Patient**								
Age(year)	35.53	11.63	−0.16	(<0.001)	−0.08	(0.120)	−0.15	(0.003)
Duration of illness	7.38	6.23	0.01	(0.728)	−0.02	(0.646)	0.02	(0.659)

MCESQ, Modified Consumer Experiences of Stigma Questionnaire; SSRS, Social Support Rating Scale.

**Table 2:** Categorical socio-demographic variable of caregivers and relevance on mean of stigma scores, discrimination scores and MCESQ scores (*N* = 427).

Variable			Mean of Stigma		Mean of Discrimination		Mean of MCESQ	
	N(%)		Mean(SD)	t/*F* (*p*)	Rank sum	Z/Chi squared with tie (*p*)	Mean(SD)	t/*F* (*p*)
**Gender**				−1.53		−0.96		−1.45
Male	222(52.0)		2.85(0.05)	(0.126)	46300.5	(0.338)	2.41(0.03)	(0.147)
Female	205(48.0)		2.96(0.05)		45077.5		2.47(0.03)	
**Marital status**				3.49		1.00		1.85
Single	30(7.0)		2.64(0.58)	(0.032)	7058.50	(0.606)	2.32(0.42)	(0.162)
Married	358(83.8)		2.91(0.71)		76142.50		2.44(0.45)	
Divorced/widowed	39(9.1)		3.09(0.77)		8177.00		2.53(0.48)	
**Kinship with patient**				8.16		6.91		7.85
Parents	233(54.6)		3.04(0.73)	(<0.001)	52154.50	(0.141)	2.52(0.45)	(<0.001)
Spouse	113(26.5)		2.88(0.64)		24240.00		2.44(0.41)	
Child	45(10.5)		2.43(0.51)		7860.50		2.14(0.38)	
Sibling	27(6.3)		2.79(0.66)		5441.50		2.35(0.43)	
Other	9(2.1)		2.67(1.18)		1681.50		2.25(0.71)	
**Education**				1.47		0.10		0.73
≤Primary	175(41.0)		2.97(0.69)	(0.223)	37333.00	(0.992)	2.46(0.42)	(0.532)
Middle school	142(33.3)		2.91(0.76)		30481.00		2.44(0.48)	
High school	75(17.6)		2.86(0.7)		15885.50		2.42(0.45)	
≥College	35(8.2)		2.7(0.62)		7678.50		2.35(0.43)	
**Employment**				0.12		2.20		0.20
Employed	269(63.0)		2.92(0.69)	(0.886)	56753.50	(0.333)	2.44(0.44)	(0.819)
Unemployed	75(17.6)		2.89(0.78)		17450.50		2.46(0.49)	
Retired	83(19.4)		2.88(0.73)		17174.00		2.41(0.46)	
**Province**				−4.56		−1.97		−3.96
* Guangdong*	229(53.6)		2.76(0.04)	(<0.001)	46528.5	(0.049)	2.36(0.03)	(<0.001)
* Zhejiang*	198(46.4)		3.08(0.05)		44849.5		2.53(0.03)	
**Settings**				2.08		1.18		1.97
Rural	228(53.4)		2.97(0.05)	(0.038 )	50282.50	(0.236)	2.48(0.03)	(0.050)
Urban	199(46.6)		2.83(0.05)		41095.50		2.39(0.03)	

MCESQ, Modified Consumer Experiences of Stigma Questionnaire.

As shown in Table 1 and Table 3, the sample consisted of patients aged between 12 and 75 years old with a mean age of 35.53(*SD* = 11.63). More than half (56.2%) of patients was female, and 43.8% were male. The majority had a low educational level (62.5%) and was unemployed (69.6%). The majority (60.7%) of patients was in the acute phase, about a quarter (24.1%) was in the remission phase, and the remainder (15.2%) was in prodromal phase. On average, the duration of illness was 7.38(*SD = *6.23) years.

**Table 3:** Socio-demographic and clinical characteristics of patients and relevance on mean of stigma scores, discrimination scores and MCESQ scores (*N* = 427).

Variable		Mean of Stigma		Mean of Discrimination		Mean of MCESQ	
	N(%)	Mean(SD)	t/*F*(*p*)	Rank sum	Z/Chi squared with tie(*p*)	Mean(SD)	t/*F*(*p*)
**Gender**			1.15		−0.48		0.87
Male	187(43.8)	2.95(0.05)	(0.25)	39422	(0.634)	2.46(0.03)	(0.384)
Female	240(56.2)	2.87(0.05)		51956		2.42(0.03)	
**Marital status**			4.27		0.71		2.69
Single	221(51.8)	3(0.71)	(0.015)	48343	(0.701)	2.49(0.43)	0.069
Married	180(42.2)	2.8(0.69)		37687		2.38(0.45)	
Divorced/widowed	26(6.1)	2.86(0.85)		5349		2.41(0.58)	
**Education level**			3.89		0.52		2.23
≤Primary	109(25.5)	2.75(0.7)	(0.009)	23431	(0.916)	2.37(0.49)	(0.084)
Middle school	158(37)	2.88(0.7)		32987		2.42(0.43)	
High School	116(27.2)	3.05(0.73)		25324		2.5(0.45)	
≥College	44(10.3)	3.05(0.65)		9637		2.52(0.4)	
**Employment**			0.99		0.53		0.57
Employed	109(25.5)	2.93(0.78)	(0.374)	24024	(0.769)	2.46(0.5)	(0.566)
Unemployed	297(69.6)	2.91(0.69)		62717		2.44(0.43)	
Retired	21(4.9)	2.7(0.71)		4638		2.34(0.47)	
**Phase of illness**			3.30		0.91		3.78
Prodromal	65(15.2)	2.94(0.72)	(0.038)	13327	(0.634)	2.45(0.46)	(0.024)
Acute	259(60.7)	2.96(0.73)		57834		2.48(0.46)	
Remission	103(24.1)	2.75(0.65)		20218		2.33(0.4)	

MCESQ, Modified Consumer Experiences of Stigma Questionnaire**.**

### 2. Descriptive analyses of stigma and discrimination

Table 4 shows the MCESQ scores for each item. The mean scores for the MCESQ scale were as follows: 2.44 for the overall MCESQ score (*SD = *0.45) (between “seldom” and “sometimes”); 2.91 for stigma experiences (*SD* = 0.71) (nearly “sometimes”); and 1.97 for discrimination experiences (*SD* = 0.37) (nearly “seldom”).

**Table 4:** Item description of MCESQ (*N* = 427).

	Item	1	2	3	4	5	*Mean*
		(%)	(%)	(%)	(%)	(%)	*(SD)*
1	I have worried that others will view me unfavorably because myfamily member receives psychiatric treatment.[S]	30.0	21.8	28.1	12.4	7.7	2.46 (1.25)
2	I have been in situations where I heard others say unfavorable oroffensive things about persons and their psychiatric disorders.[S]	39.1	24.8	26.7	7.0	2.3	2.09 (1.07)
3	I have seen or read things in the mass media (e.g., television, movies,books) about persons receiving psychiatric treatment and theirpsychiatric disorders which I find hurtful or offensive.[S]	53.4	21.8	18.7	4.5	1.6	1.79 (1)
4	I have avoided telling others outside of my immediate family that myfamily member has received psychiatric treatment.[S]	15.9	19.2	17.3	22.3	25.3	3.22 (1.42)
5	I have been treated as less competent by others when they learned myfamily member had received psychiatric treatment.[S]	21.8	31.6	27.2	12.2	7.3	2.52 (1.17)
6	I have been shunned or avoided by others when they learned myfamily member received psychiatric treatment.[S]	33.5	28.6	23.4	10.1	4.5	2.23 (1.15)
7	I have been lower my expectations for accomplishmentsin life becausemy family member receives psychiatric treatment.[S]	19.2	18.5	36.8	19.4	6.1	2.75 (1.15)
8	I have been treated fairly by others when they knewmy family receive psychiatric treatment.[S]^*^	19.4	29.3	27.4	20.1	3.8	2.59 (1.22)
9	My friends are understanding and supportive after learning that my family member receive psychiatric treatment.[S]^*^	18.0	23.4	29.3	19.4	9.8	2.80 (1.23)
10	I have been avoided indicating on written applications (for jobs,licenses, housing, school, etc.)that my family received psychiatrictreatment for fear that information would be usedagainst me or my family.[D]	37.9	24.1	19.2	9.8	8.9	2.28 (1.3)
11	I have been treated with kindness and sympathy by government officials when they learned my family received psychiatric treatment.[D]*	60.4	16.2	14.5	5.9	3.0	1.75 (1.09)
12	Co-workers or supervisors at work have been supportiveand accommodating when they learn that my familymember received psychiatric treatment.[D]*	47.8	13.8	17.8	14.3	6.3	2.18 (1.33)
13	I have been turned down for a job, for which I were qualified,when it was learned my family member receivedpsychiatric treatment.[D]	78.2	11.9	6.3	3.0	0.5	1.36 (0.77)
14	I have been excluded from social activities when it was knownI had a family member received psychiatric treatment.[D]	78.9	14.3	4.0	2.3	0.5	1.31 (0.7)
15	I have had the fact that my family received psychiatrictreatment used against me in legal proceedings.[D]	88.8	8.0	1.4	1.2	0.7	1.17 (0.57)
16	I have had difficulty in renting an apartment or findingother housing when my family member’s illness was known.[D]	86.4	8.7	4.0	0.5	0.5	1.2 (0.57)
17	I have been denied educational opportunities when it waslearned that my family member receivedpsychiatric treatment.[D]	86.4	9.1	3.5	0.7	0.2	1.19 (0.54)
18	I have been denied a business license, temporary residence permits,driver’s license, or other kind of permit when it was learned Ihad a family member received psychiatric treatment.[D]	89.7	7.7	1.9	0.5	0.2	1.14 (0.46)
	Score of stigma						2.91(0.71)
	Score of discrimination						1.97(0.37)
	Total score of MCESQ						2.44(0.45)

1.1 = never, 2 = seldom, 3 = sometimes, 4 = often, 5 = very often,

2.* means reverse item,

3.[S] means stigma; [D] means discrimination.

The mean score of “I have avoided telling others outside of my immediate family that my family member has received psychiatric treatment” was above 3, and 64.9% answered “sometimes” to “always”. This finding indicates that caregivers themselves would like to conceal the fact that a family member was receiving treatment for mental illness. Moreover, 70.7% of caregivers lacked the support of friends. The mean score for “friends understanding and supportive” was 2.80, showing that caregivers lacked understanding and support from friends. Additionally, because of their family situation, more than half (62.3%) of caregivers had lowered their expectations for accomplishments in life. Nearly half (48.2%) of caregivers reported that they had been unfavorably treated and 38.0% had shunned/avoided by others who knew about their family member’s illness. Furthermore, 36.0% of caregivers had heard others saying unfavorable or offensive things about their family members. However, caregivers of persons with schizophrenia seldom experienced discrimination when watching news in the mass media, renting a house, searching for a job, acquiring education, joining social activities, applying for a license, in legal proceedings and in dealing with government officials. The most frequent areas in which discrimination occurred while working with co-workers and supervisors (38.4%) and writing paper application for jobs, licenses, housing, school and so on (37.9%).

### 3. Bivariate correlations

Table 1, 2 and 3 present the bivariate analyses for stigma experience mean scores, discrimination experience mean scores, and MCESQ mean scores with possibly associates.

Pearson analysis found the age of caregivers was significantly positively related to stigma (*r = 0.12*, *p*<0.05). Different family kinship with patients also associated with caregivers’ experience of stigma. As demonstrated in Table 2, parents and spouses suffered significantly more stigmas than siblings, children and other family members (*F* = 8.16, *df* = 4, *p*<0.001) and children experienced fewest. Additionally, the relationship between kinship and MCESQ scores was also statically significant (*F* = 7.85, *df* = 4, *p*<0.001). Besides, their living areas were also related to their stigma experience and the total MCESQ scores. As showed, the mean stigma scores of *Zhejiang* was 3.08 (*SD* = 0.05), significantly more than that in *Guangdong* (*Mean* = 2.76, *SD* = 0.04, t = 4.56, *p*<0.001). Caregivers from rural areas also reported more stigma experiences than those from urban (t = 2.08, *p*<0.05). SSRS scores were negatively associated with stigma experience (r = −0.24, *p*<0.001) and MCESQ scores (r = −0.21, *p*<0.001). However, caregivers’ gender, educational level, marital status, and family income were not statically significantly related to MCESQ scores.

The bivariate analysis suggested that the stigma experience of caregivers was negatively correlated with patient age (r = −0.16, *p*<.001), educational level (*F* = 3.89, *df* = 3, *p*<0.01) and related to marital status (*F* = 4.27, *df* = 2, *p*<0.05) and phase of illness (*F* = 3.30, *df* = 2, *p*<0.05). Caregivers experienced more stigmas when patients were young, singled, highly educated and in the acute phase. Patient gender, employment status and duration of disease did not appear to influence caregivers’ experience of stigma. Similarly, mean score of MCESQ was negatively related to patients age (r = −0.15, *p*<0.01) and associated with phase of illness (*F* = 3.78, *df* = 2, *p*<0.05).

The caregivers’ experience of discrimination was only significantly correlated with province (*Z* = −1.97, *p*<0.05). However, the relations between discrimination and other patient and self-factors were not significant.

### 4. Regression Analyses

Three models (see Table 5) were investigated in this article, one for the caregivers’ stigma experience score (Model 1), one for the caregivers’ discrimination experience score (Model 2) and another for the caregivers’ MCESQ scores (Model 3). Models included only those associate variables that were related with the relevant stigma score, discrimination score and MCESQ score at p<0.05. The first and third models yielded significant overall probability levels, accounting for 14.2% and 10.5% of variance in the dependent variables. Candidate factors for the three multivariate models were selected on the basis of their significant partial correlation with the corresponding scores (Table 1, Table 2 & Table 3). Model 1 and Model 3 indicated that the degree of caregivers’ social support was significantly negative related to the caregivers’ stigma (standard β = −0.20, *p*<0.001) and total MCESQ scores (std. β = −0.17, *p*<0.001). Furthermore, children of patients suffered significantly fewer experiences of stigma than spouses, parents and other relatives who were caregivers (std. β = −0.18, *p*<0.001). In addition, if patient did not or only complete primary school, the caregivers experienced fewer stigmas (std. β = −0.13, *p*<0.01). Moreover, people living in urban areas experienced fewer stigmas (std. β = −0.12, *p*<0.01). Furthermore, caregivers from *Zhejiang* reported more stigmas (std. β = 0.15, *p*<0.005), discrimination (unadjusted B = 0.67, *p*<0.05) and MCESQ scores (std. β = 0.13, *p*<0.01) than people from *Guangdong.*

**Table 5:** Regression analysis of caregiver and patient characteristics on caregivers’ stigma experience, discrimination experience and MCESQ scores in the latest month. (N = 427).

Variable	Model 1: Stigma				Model 2: Discrimination			Model 3: Total MCESQ scores			
	B	SE	*p*	β	B	SE	*p*	B	SE	*p*	β
Child	−3.82	0.96	<0.001	−0.18				−5.10	1.23	<0.001	−0.19
≤Primary School(patient)	−1.93	0.67	0.004	−0.13							
Urban	−1.61	0.59	0.007	−0.12				−1.66	0.75	0.027	−0.10
Zhejiang	1.93	0.61	0.002	0.15	0.67	0.30	0.023	2.08	0.78	0.008	0.13
SSRS	−0.18	0.04	<0.001	−0.20				−0.19	0.05	<0.001	−0.17
(Constant)	32.09	1.34	<0.001		17.08	0.20		49.73	1.69	<0.001	
R^2^		0.152							0.114		
Ad R^2^		0.142							0.105		
F		15.10				5.19			13.51		
Sig.		<0.001				0.023			<0.001		

1.Model 1: multiple linear regression for stigma subscale scores; Model 2: robust regression for discrimination subscale scores; Model 3: multiple linear regression for total MCESQ scores,

2.MCESQ, Modified Consumer Experiences of Stigma Questionnaire; SSRS, Social Support Rating Scale,

3.Kinship: caregiver was the child of the patient = 1, parents/kin/sibling/others = 0; Patient’s educational level: ≤primary school = 1, >primary school = 0; Caregiver’s residence area: urban = 1, rural = 0; Caregiver’s province: *Zhejiang* = 1, *Guangdong* = 0.

## Discussion

This study found that the family caregivers of patients with schizophrenia generally experienced stigmas in daily life and discrimination experiences are comparably fewer. This finding was based on the actual reported experiences of caregivers rather than answers to questions about hypothetical situations. Our results showed that caregivers usually avoided telling others that their family members were receiving schizophrenia treatment. Furthermore, caregivers felt that it was difficult to obtain support from friends. Experiences of discrimination among caregivers were rare, especially in terms of housing, interactions with officials, license applications, education and social activities. Additionally, some caregivers thought they were discriminated against in the workplace. During China’s planned economy period, it was “work unit” (*Danwei*) that provided all the welfare to families and individuals, including wages, schooling, housing, etc., thus there are a lot of people who still want to seek help from work unit to solve the problem of many families [34]. Such phenomena still exist. For example, when family member suffer from serious illness, caregivers will apply to his/her enterprises and institutions for special economic aids or caring leaves. To our experience, it is hard to conceal a family member’s mental illness from employers, because caregivers have to explain the reasons when they ask for leave. Similar situations occurred when caregivers were filling out application forms (for jobs, licenses, housing, school, etc.). Caregivers worried that disclosing a family member’s mental illness might hurt their family member or themselves. A lack of trust in professionals could result in a fear of disclosing information.

Our study also found that this type of experience of stigma and discrimination is associated with caregivers’ social support, kinship, patients’ education level and living areas.

First, we posited that social support was significantly related to caregivers’ experiences of stigma. People with high levels of social support experienced fewer stigmas. This broadens Mueller et al.’s finding that social support can modify perceived stigmatization in mental ill group [35]. Informal and formal social support from mental health professionals and other families can play a positive role in care-giving and help caregivers overcome negative information, get through difficult times and draw on inner strength [36]. At the same time, caregivers with high social support most likely live in a friendly and open environment. Other people give the caregivers helps, understanding and assistances rather than looking down on them or delivering socially stigmatizing information.

Second, in our study, we also found that kinship of caregivers with patients related with experiences of stigma. In particular, children perceived fewer stigmas than other family caregivers. Existing research has found that parents, especially mothers, are the main caregivers of adult family members who have schizophrenia [37]. Corrigan and Miller’s review suggested that parents often blamed themselves for causing their child’s illness [38]. Our study also demonstrated that half of people with schizophrenia were cared for by parents. Different *Guanxi* (social network) have different degrees of influence on one’s daily life, which is well-known as *Chaxu geju* (the differential mode of association) [39]. Because of traditional culture, Chinese parents always consider children as members of their family, even if they are married [40]. In China, mental illness often harmed family honor and was associated with “loss of face” [41]. Presently, Chinese parents suffer increased distress and pressure because of the one-child policy [42]. However, with reduced family sizes, more and more children live away from their parents after marriage, making it easier for a child to keep a parent’s severe mental illness secret than it is for a parental caregiver to keep a child’s mental illness secret. Except that, young adult receive more education and often leave their hometown to work in brand-new area. Therefore, adult children of person with mental illness experienced fewer stigmas.

Third, patients’ education level was another relative factor. Caregivers reported significantly more stigma experience when their family member with schizophrenia was highly educated. Our finding is consistent with previous findings [18], [22]. This result might be explained in this way: education level is one of most important predictors for one’s social and economic status. Also, social culture and traditional norm give more expectation to people who perceived highly education. Once the abnormal situation occurred, the gaps between reality and expectation may make the family members become more sensitive to others’ attitude and behaviors and feel “lose face”.

Fourth, the urbanization and residence province also associated with their stigma and discrimination experience. The region and setting can capture the factors which are hard to measure, like culture, law, institution in local areas. Cultures in *Guangdong* (*Lingnan* Culture) and *Zhejiang* (*Wuyue* Culture) provinces both appreciate family values and clan traditions [43], and the two areas have similar levels of economic development (in terms of GDP), but the difference was significant. In our study, caregivers had more stigma and discrimination experience in *Zhejiang* province and rural areas. This kind of regional disparity also reveals that objective living environment is important associates of stigma and discrimination experience.

It was also needed to pay attention to the association between stigma experience with the phase of illness and the age of caregivers and patients, although their relations were only statistically significant in bivarite analysis. Caregivers experienced more stigmas in the patients’ acute phase in which patients’ abnormal behavior is frequently and easily observed by neighbors, friends and other people. Therefore, stigma will be more readily experienced by such caregivers. Then, caregivers’ age was positively associated with caregivers’ experiences of stigma and patients’ age was negatively related to stigma experiences. It echoes the result in other research that the elder group encountered more discrimination because elder caregivers might provide a long period of care for their adult children [44] or other relatives; they are also more sensitive to stigma from others. Furthermore, stigma led to a poor quality of life for long-time caregivers [1]. Therefore, a lifelong perspective regarding caregivers’ experiences is necessary.

Finally, there was no evidence from our study to support the idea that gender, marital status, duration of illness or caregiver’s education level had relationship with experiences of stigma or discrimination in the target area of China. We did not observe any gender differences in scores of stigma and experiences of discrimination, similar to previous research [23]. Additionally, although half of patients were single, marital status did not affect caregivers’ experiences of stigma in the regression analysis. One possible explanation for the lack of a correlation between caregivers’ educational level and experiences of stigma or discrimination is that most participates in our samples did not or just complete middle school. Furthermore, duration of the illness was not related to the stigma and discrimination experience in the short period which might be explained that schizophrenia is variable and chronic.

Besides, it is important to highlight four possible limitations of the present study. First of all, we conducted our research in *Zhejiang* and *Guangdong* provinces, two of the most economically developed areas in China. Therefore, our results may not represent the stigmatization of all caregivers in China, considering variations in ethnic groups and regional cultures. Second, the MCESQ was originally used among consumers or persons with mental illness. However, the reliability and validity of the modified scale in our study appeared to be good. Third, the three models in our study were based on the results of bivariate correlation analysis rather than on theory, so they might not apply to other samples, at the same time, it cannot reveal causal relation. Fourth, we conducted face-to-face structured interview rather than fill questionnaires by themselves in the data collection procedure, which may lead to a social desirability bias.

## Conclusions

Caregivers of people with schizophrenia experienced considerable stigmas, which were related to social support, kinship, patients’ educational level and regional factors. Discrimination was rarely reported by caregivers, but province difference was significant. It is suggested that more social support should give to those caregivers whose family members are suffering from severe mental illness.

## References

[1] Chou YC, Pu CY, Lee YC, Lin LC, Kröger T (2009) Effect of perceived stigmatisation on the quality of life among ageing female family carers: A comparison of carers of adults with intellectual disability and carers of adults with mental illness. J Intell Disabil Res 53: 654–664. 10.1111/j.1365-2788.2009.01173.x 19490349

[2] Mak WWS, Cheung RYM (2011) Psychological distress and subjective burden of caregivers of people with mental illness: The role of affiliate stigma and face concern. Community Ment Health J 48: 270–274. 21681460 10.1007/s10597-011-9422-9

[3] Corrigan P, Thompson V, Lambert D, Sangster Y, Noel JG, et al (2003) Perceptions of discrimination among persons with serious mental illness. Psychiat Serv 54: 1105–1110. 10.1176/appi.ps.54.8.1105 12883137

[4] Gonza Lez-Torres MA, Oraa R, Arı Stegui M, Ferna Ndez-Rivas A, Guimon J (2007) Stigma and discrimination towards people with schizophrenia and their family members: A qualitative study with focus groups. Soc Psychiatry Psychiatr Epidemiol 42: 14–23. 17036263 10.1007/s00127-006-0126-3

[5] Lv Y, Wolf A, Wang X (2013) Experienced stigma and self-stigma in Chinese patients with schizophrenia. Gen Hosp Psychiat 35: 83–88. 10.1016/j.genhosppsych.2012.07.007 22925273

[6] Goffman E (1963) Stigma: Notes on the management of spoiled identity. New York: Simon and Schuster. p141.

[7] Kleinman A, Kleinman J (1993) Face, favour and families: The social course of mental health problems in Chinese and American societies. Chinese J of Ment Health 6: 37–47.

[8] Struening EL, Perlick DA, Link BG, Hellman F, Herman D, et al (2001) Stigma as a barrier to recovery: The extent to which caregivers believe most people devalue consumers and their families. Psychiat Serv. 52: 1633–1638. 10.1176/appi.ps.52.12.1633 11726755

[9] Muhlbauer S (2002) Experience of stigma by families with mentally ill members. J Am Psychiatr Nurses Assoc 83: 76–83.

[10] Karnieli-Miller O, Perlick DA, Nelson A, Mattias K, Corrigan P, et al (2013) Family members’ of persons living with a serious mental illness: Experiences and efforts to cope with stigma. J Ment Health 22: 254–262. 23662789 10.3109/09638237.2013.779368

[11] Larson JE, Corrigan P (2008) The stigma of families with mental illness. Acad Psychiatry 32: 87–91. 18349326 10.1176/appi.ap.32.2.87

[12] Karamlou S, Mottaghipour Y (2013) Cultural understanding of stigma: A qualitative study in families of patients with severe psychiatric disorders in Iran. Eur Psychiat 28 Supplement 1 1.

[13] Snowden LR, Yamada A (2005) Culture differences in access to care. Annu Rev Clin Psychol 1: 143–166. 17716085 10.1146/annurev.clinpsy.1.102803.143846

[14] Lin K, Kleinman AM (1988) Psychopathology and clinical course of schizophrenia: A cross-cultural perspective. Schizophr Bull 14: 555–567. 3064282 10.1093/schbul/14.4.555

[15] Pearson V (1993) Families in China: An undervalued resource for mental health? J of Fam Ther 15: 163–185.

[16] Kung WW (2003) The illness, stigma, culture, or immigration? Burdens on Chinese American caregivers of patients with schizophrenia. Fam Soc 84: 547–557.

[17] Kirmayer LJ (1989) Cultural variations in the response to psychiatric disorders and emotional distress. Soc Sci Med 29: 327–339. 2669146 10.1016/0277-9536(89)90281-5

[18] Phillips MR, Pearson V, Li F, Xu M, Yang L (2002) Stigma and expressed emotion: A study of people with schizophrenia and their family members in China. Br J Psychiatry 181: 488–493. 12456518 10.1192/bjp.181.6.488

[19] Lee S, Lee MTY, Chiu MYL, Kleinman A (2005) Experience of social stigma by people with schizophrenia in Hong Kong. Br J Psychiatry 186: 153–157. 15684240 10.1192/bjp.186.2.153

[20] Gao S, Philips M, Wang X, Xu D, Jia Z, et al (2005) Experience of stigma among patients with schizophrenia and their family members and attitudes of different groups about this stigma. Chinese Ment Heal J 19: 82–86.

[21] Girma E, Moller-Leimkuhler AM, Dehning S, Mueller N, Tesfaye M, et al (2014) Self-stigma among caregivers of people with mental illness: Toward caregivers’ empowerment. J Multidiscip Healthc 7: 37–43. 24470760 10.2147/JMDH.S57259 PMC3896287

[22] Phelan JC, Bromet EJ, Link BG (1998) Psychiatric illness and family stigma. Schizophr Bull 24: 115–126. 9502550 10.1093/oxfordjournals.schbul.a033304

[23] Shibre T, Negash A, Kullgren G, Kebede D, Alem A, et al (2001) Perception of stigma among family members of individuals with schizophrenia and major affective disorders in Ethiopia. Soc Psychiatry Psychiatr Epidemiol 36: 299–303. 11583460 10.1007/s001270170048

[24] Charles H, Manoranjitham SD, Jacob KS (2007) Stigma and explanatory models among people with schizophrenia and their relatives in Vellore, south India. Int J Soc Psychiatry 53: 325–332. 17703648 10.1177/0020764006074538

[25] Phillips MR, Li Y, Stroup TS, Xin L (2000) Causes of schizophrenia reported by patients’ family members in China. Br J Psychiatry 177: 20–25. 10945083 10.1192/bjp.177.1.20

[26] Magaña SM, Ramírez GJI, Hernández MG, Cortez R (2007) Psychological distress among latino family caregivers of adults with schizophrenia: The roles of burden and stigma. Psychiatr Serv 58: 378–384. 17325112 10.1176/appi.ps.58.3.378 PMC2396526

[27] Chang KH, Horrocks S (2006) Lived experiences of family caregivers of mentally ill relatives. J Adv Nurs 53: 435–443. 16448486 10.1111/j.1365-2648.2006.03732.x

[28] Yang LH, Purdie-Vaughns V, Kotabe H, Link BG, Saw A, et al (2013) Culture, threat, and mental illness stigma: Identifying culture-specific threat among Chinese-American groups. Soc Sci & Med 88: 56–67. 23702210 10.1016/j.socscimed.2013.03.036 PMC4043281

[29] Wahl OF (1999) Mental health consumers’ experience of stigma. Schizophr Bull 25: 467–478. 10478782 10.1093/oxfordjournals.schbul.a033394

[30] Dickerson FB, Sommerville J, Origoni AE, Ringel NB, Parente F (2002) Experiences of stigma among outpatients with Schizophrenia. Schizophr Bull 28: 143–155. 12047014 10.1093/oxfordjournals.schbul.a006917

[31] Brohan E, Slade M, Clement S, Thornicroft G (2010) Experiences of mental illness stigma, prejudice and discrimination: A review of measures. Health Service Research 10: 80–91. 10.1186/1472-6963-10-80 PMC2851715 20338040

[32] Livingston JD, Boyd JE (2010) Correlates and consequences of internalized stigma for people living with mental illness: A systematic review and meta-analysis. Soc Sci & Med 71: 2150–2161. 21051128 10.1016/j.socscimed.2010.09.030

[33] Xiao S (1994) Theoretical basis and research applications of Social Support Rating Scales (SSRS). J of clin psychiatr [Chinese] 4: 98–100.

[34] Saunders P, Shang X (2001) Social security reform in china’s transition to a market economy. Social Policy & Administration 35: 274–289.

[35] Mueller B, Nordt C, Lauber C, Rueesch P, Meyer PC, et al (2006) Social support modifies perceived stigmatization in the first years of mental illness: A longitudinal approach. Soc Sci & Med 62: 39–49. 15992979 10.1016/j.socscimed.2005.05.014

[36] Chen F, Greenberg JS (2004) A positive aspect of caregiving: The influence of social support on caregiving gains for family members of relatives with schizophrenia. Community Ment Health J 40: 423–435. 15529476 10.1023/b:comh.0000040656.89143.82

[37] Chafetz L, Barnes L (1989) Issues in psychiatric caregiving. Arch Psychiat Nurs 3: 61–68. 2712593

[38] Corrigan PW, Miller FE (2004) Shame, blame, and contamination: A review of the impact of mental illness stigma on family members. J of Ment Health 13: 537–548.

[39] Fei X (1992) From the soil: The foundations of Chinese society. California: University of California Press.

[40] Wu F (2010) Suicide and Justice: A Chinese perspective. Oxon: Routledge Press.

[41] Sue DW (1994) Asian-American mental health and help-seeking behavior. J Couns Psychol 21: 167–178.

[42] Miao C, Li C, Zhong G, Yuan C, Wang L, et al (2003) Mental health status of parents who has only one mentally ill child. Chinese J of Clinical Rehabilitation 7: 3384.

[43] Xiang B (2000) Transcending boundaries: Zhejiangcun: The story of a migrant village in Beijing [Chinese]. Beijing: SDX Joint Publishing Company. p 454–457.

[44] Kelly TB, Kropf NP (1995) Stigmatized and perpetual parents: Older parents caring for adult children with life-long disabilities. J Gerontol Soc Work 24: 3–16.

